# Prevention of Tuberculosis*Read before the Massachusetts Veterinary Association, Jan., 1894.

**Published:** 1894-03

**Authors:** John M. Parker


### PREVENTION OF TUBERCULOSIS.*

By John M. Parker, D. V. S.

Believing that the State Veterinary Association ought to lead in formulating laws for the attempted prevention of tuberculosis, I have endeavored, in a rough way, to bring the matter before the Association, so that, if thought advisable, some action might be taken on the matter.

At the present time prophylactic measures for the purpose of controlling and checking the spread of tuberculosis are attracting a great deal of attention, not only from the veterinary profession, but medical associations and State boards of health throughout the country are taking the matter up, and in several States the health officials have placed tuberculosis on the list of contagious diseases, and otherwise have taken decided action on the matter.

Michigan was the first to adopt this line of action, and on Sept. 30, 1893, "The Michigan State Board of Health unanimously decided that consumption is a dangerous, communicable disease, and must be reported by physicians and householders to the several Boards of Health." (Med. Record, Nov. 18, 1893.)

Pennsylvania next came into line, and in Oct. the "Philadelphia County Medical Society" appealed to the Board of Health of Philadelphia to place tuberculosis upon the list of contagious diseases to be reported to the Board.

The Health Department of the State of New York, acting on the advice of Dr. H. M. Biggs, the Bacteriologist of the Department, has taken a similar position. (See p. 832 Med. Record, Dec. 23, 1893.)

While at Chicago, the American Public Health Association, in conjunction with the International Congress of Public Health, adopted the following practical recommendations for the prevention of disease:

1. The notification and registration by health authorities of all cases of tuberculosis which have arrived at the infectious stage.
2. The thorough disinfection of all houses in which tuberculosis has occurred, and the recording of such action in an open record.
3. The establishment of special hospitals for the prevention of tuberculosis.
4. The organization of societies for the prevention of tuberculosis.

*Read before the Massachusetts Veterinary Association, Jan., 1894.

5. Government inspection of dairies and slaughter-houses, and the extermination of tuberculosis among dairy cattle.

6. Appropriate legislation against spitting into places where the sputum is liable to infect others, and against the sale or donation of objects which have been in use by consumptives, unless they have been thoroughly disinfected.

7. Compulsory disinfection of hotel rooms, sleeping-car berths and steamer cabins which have been occupied by consumptives, before other persons are allowed to occupy them.

The reports and recommendations of the Committee were adopted.

Massachusetts ought not to be far in the rear, and to give credit where it is due, I believe at least one of the local inspectors in Massachusetts has advised the health officers of his locality to place tuberculosis on the list of contagious diseases. (I refer to a report by Dr. Winchester, of Lawrence, to the local Board of Health.)

In Massachusetts the cattle commissioners are an independent commission, having no connection with the Board of Health, and so far there has been no adequate legislation sufficient to check the spread of tuberculosis among our dairy stock. The same applies, though in a less degree, to the action taken by the State of N. Y., for it is not enough to isolate and destroy infected cattle: *such action alone does not strike at the root of the matter. Hitherto the influence exerted by the conditions and surroundings under which the animals are kept has been almost entirely overlooked.*

Seed always requires a proper soil before it will develop. The bacilli requires a suitable culture media, and we know that tubercle bacilli is one of the most exacting in this respect.

It is not sufficient that the bacilli should be inhaled; we know that all animal bodies in a healthy condition have a great disease-resisting power, and it requires more than the mere presence of the bacteria to cause the disease. In other words, the bacteria is only a small part of the disease. As a contributor to the Med. Record (Sept. 2, 1893) truly says, "There are other factors equally as important in the causation of the malady as the part played by the microbe."

In the report of the Congress for the study of Tuberculosis held in Paris in 1891, it is said, "If there is one thing above all others that is learned from the many discussions upon this well-worn but still obscure subject of tuberculosis, it is that the bacilli alone does not cause the disease. The host is obviously not the least important element."

We must all acknowledge that there is in the constitution of the individual before bacilliary infection takes place, something that determines the fact of infection, and that largely determines the course and character of the results of infection. Parasites thrive only under certain conditions. Remove the conditions which are favorable for their propagation, and they disappear. "You take away their lives when you take away the means by which they live," has been well said. In other words, build up the health and constitution of the animal body, increase the disease-resisting powers, and you are taking a long step in the right direction. On the other hand, pay no attention to the general health of the animal, allow its general health to run down, weaken its constitution by breeding young and breeding continuously, force the milk supply, and continue milking while the cow is in calf, feed high and give little exercise, keep in a close, hot barn in unsanitary surroundings, and you produce an animal that is just in the right condition to contract disease, yet these are exactly the conditions usually found in the average dairy farm.

Breeders and farmers pay little or no attention to the general health and constitution of their dairy stock. They seem to consider excessive milking qualities to be the sole aim and purpose of breeding. I had a good illustration of this the other day. My attention was called to a heifer, 14 months old. It was strong and rugged for its age. The owner asked me what I thought of breeding her when she came in heat ; a farmer standing near by remarked that it was the best thing he could do. I asked him why. He said because she would become a better milker. That, I believe, is a fair sample of the way farmers look on the matter. He would have her a big milker at whatever cost to her strength and constitution.

Again, what is to be said about the practice of continuing to milk a cow while carrying a calf? Through use and wont it has become the invariable practice, at the same time I believe it to be one of the most serious of the many serious conditions that tend to sap the strength and vitality of our dairy cattle.

Probably you are aware what the result would be if a woman were to nurse a child while pregnant, yet not only is this invariably done in dairy cows, but the milking qualities are forced to the utmost limit, and if she gets a month's rest before calving, she is in luck. On top of this, inbreeding is commonly practiced, and a cow is expected to bear a calf with unfailing regularity year after year, and if unable to do so, she is thrown aside as worthless.

Nor is this all—all of you are familiar with the hot, foul air, reeking with the smell of the cattle, that will meet you when the barn door is opened in the morning. The cows in many cases are packed in, every crevice and opening through which the air may gain admittance is blocked up, and they are kept in these conditions during the entire winter, with little or no exercise. *You have here just the conditions that are most suited for the development of the disease.*

Introduce a consumptive cow into a herd kept in this way. The cow has a cough possibly, or more or less of a discharge. The discharge comes in contact with the woodwork. This dries up, particles fall off and mix with the hay and dust and chaff. The barn is close and stuffy, the animals close together, there is no fresh air to carry away the poison-laden atmosphere. They breathe the same air over and over again, *with their noses in the most dangerous place, close to the ground, where the dust and dirt accumulate.* What wonder if the whole herd, or a large percentage of them, become diseased in a short space of time!

And it is not only in the ventilation. What about the manure heaps; what about the slime and filth so often heaped up in the cellar below; what about the water supply? The cattle often get their water supply from what is practically little better than a cesspool.

*Are these animals living in healthy surroundings? Is it common sense to expect them to be strong and healthy and free from disease?*

To counteract this state of affairs, the Cattle Commissioners have been in the habit of taking *only* the pronounced cases and destroying them, and leaving animals that are predisposed to disease, animals that are weak constitutionally, not to mention those infected animals that they have failed to discover, and leaving them right in the midst of infected surroundings. The result is what one would naturally expect: little or no progress has been made in eradicating the disease, and if no change is made in the system at present in use, their work might be continued indefinitely without any practical result.

Instead of being in the hands of an independent board, the supervision of the matter should be in the hands of the State Board of Health. Of course, it is not possible for any great change to be made at a jump, but it is possible for a system of education to be begun at once, always keeping in view the ultimate adoption of a more perfect system of State supervision and inspection of dairy farms.

Any proper system of inspection should embody the following points:

I. The whole matter should be transferred to the State Board of Health. The Board of Agriculture shall be expected to co-operate by spreading information on the subject among farmers and stock-raisers by means of lectures and pamphlets, as has been done in Pennsylvania and other States.

II. The State should be divided into districts, each district to be in the charge of a competent veterinary surgeon as inspector, who shall be responsible only to the Chief State Inspector.

III. One or more quarantine stations shall be established in each district, so that suspicious cases, or cases to be kept in quarantine, may be sent there to be kept or killed, as is thought fit.

IV. Owners of cattle killed shall be compensated by the State.

V. No person shall be allowed to sell milk without a license, the license to be granted by the State, after a satisfactory examination of the dairy and herd by the Inspector in the district.

The Inspector's duties shall embrace the following points:

1. He shall make periodical visits to all the licensed dairies in his district.

2. He shall condemn all tuberculous cattle; suspicious cases shall be tested with tuberculin, and, if it is thought proper, sent to the quarantine station of the district, to be killed or otherwise, as thought fit. The owner may have the privilege of keeping suspicious cases, under certain restrictions, on the premises for the purpose of turning into beef, if he so desires. After the removal of a tuberculous animal, the Inspector shall see that the box or stall is thoroughly disinfected.

3. He shall condemn as unfit for dairy purposes all animals in an unthrifty or sickly condition.

4. He shall not grant licenses to dairies in a dirty or unsanitary condition. In this connection the following points should be noticed:

(*a*) The barns must have a minimum of at least 600 cubic feet for each animal, no height of over 15 feet to be included. (The cubic capacity to be greater if thought advisable.)

(*b*) They must have sufficient ventilation, to the reasonable satisfaction of the Inspector.

(*c*) The barn must be light, to the reasonable satisfaction of the Inspector.

(*a*) The floor of the barn must be tight, so that the urine will not soak through and drip onto the ground or into the cellar below.

(*e*) The manure shall not be allowed to lie in piles against the barn walls, but shall be a suitable distance from the farm buildings.

(*f*) The water shall be of good quality, and shall not be taken from wells in or near the barnyard, nor from wells so situated as to allow the surface drainage to flow into them.

Of course, as before remarked, it would be impossible to enforce all those requirements throughout the State at short notice, but the spirit of the instructions could be carried out and gradual changes made, the Boards of Health and the Inspectors always keeping in view the ultimate end to be attained.

In other States the Boards of Health have gone to work in a business-like way, in attempting to control tuberculosis in the human being; why cannot the State of Massachusetts do the same with her dairy cattle? I believe the time has come when something definite should be done. No better time could be chosen than the present, and with this end in view, I move "That a committee be appointed to report to this Association on the matter, so that the Association will be able to communicate with the State Medical Society and the State Board of Health, inviting their presence and assistance in discussing intelligently the best method of controlling and checking the spread of bovine tuberculosis."

